# Hantaviruses and TNF-alpha act synergistically to induce ERK1/2 inactivation in Vero E6 cells

**DOI:** 10.1186/1743-422X-5-110

**Published:** 2008-09-29

**Authors:** Tomas Strandin, Jussi Hepojoki, Hao Wang, Antti Vaheri, Hilkka Lankinen

**Affiliations:** 1 Department of Virology, Haartman Institute, P.O. Box 21, FI-00014, University of Helsinki, Finland

## Abstract

**Background:**

We have previously reported that the apathogenic Tula hantavirus induces apoptosis in Vero E6 epithelial cells. To assess the molecular mechanisms behind the induced apoptosis we studied the effects of hantavirus infection on cellular signaling pathways which promote cell survival. We previously also observed that the Tula virus-induced cell death process is augmented by external TNF-α. Since TNF-α is involved in the pathogenesis of hantavirus-caused hemorrhagic fever with renal syndrome (HFRS) we investigated its effects on HFRS-causing hantavirus-infected cells.

**Results:**

We studied both apathogenic (Tula and Topografov) and pathogenic (Puumala and Seoul) hantaviruses for their ability to regulate cellular signaling pathways and observed a direct virus-mediated down-regulation of external signal-regulated kinases 1 and 2 (ERK1/2) survival pathway activity, which was dramatically enhanced by TNF-α. The fold of ERK1/2 inhibition correlated with viral replication efficiencies, which varied drastically between the hantaviruses studied.

**Conclusion:**

We demonstrate that in the presence of a cytokine TNF-α, which is increased in HFRS patients, hantaviruses are capable of inactivating proteins that promote cell survival (ERK1/2). These results imply that hantavirus-infected epithelial cell barrier functions might be compromised in diseased individuals and could at least partially explain the mechanisms of renal dysfunction and the resulting proteinuria seen in HFRS patients.

## Background

Hantaviruses (Family *Bunyaviridae*, Genus *Hantavirus*) are viruses which chronically infect rodents and insectivores with no apparent disease but in humans they cause two major clinical symptoms: HFRS in Eurasia and hantavirus cardiopulmonary syndrome (HCPS) in the Americas. Some hantaviruses also seem to be apathogenic, including Tula (TULV) and Topografov (TOPV) virus [1,2]. Depending on the causative virus, HFRS manifests as mild (Puumala virus; PUUV), moderate (Seoul virus; SEOV) or severe disease (Hantaan virus; HTNV). Hantaviruses are negative-sense single-stranded RNA viruses with a tripartite genome of large (L), medium (M) and small (S) segments encoding the RNA-dependent RNA polymerase, the envelope precursor protein of two glycoproteins Gn and Gc, and the nucleocapsid protein N [3].

The multi-organ hantaviral disease is characterized by local induction of cytokines but their role in the mechanisms of pathogenesis is still poorly understood. Tumor necrosis factor-α (TNF-α) is a pro-inflammatory cytokine associated with hantavirus infections *in vivo*. Elevated TNF-α levels are found in plasma of HFRS [4,5] and HCPS [6] patients and TNF-α has been detected directly in the kidneys of NE patients [7]. TNF-α is implicated in the pathophysiology of, for example, septic shock and is capable of inducing adult respiratory distress syndrome (ARDS) in experimental animals and humans. The strong similarity of these effects to the manifestations in hantavirus diseases [8], together with the evidence of association of TNF-α polymorphism of high-producer haplotype in the severe course of PUUV infection [9], makes TNF-α a factor in hantavirus pathogenesis which deserves further attention. TNF-a is a conditional death inducer with pro-apoptotic capacity only uncovered when cell survival mechanisms are hindered. TNF-α-induced programmed cell death occurs via the cleavage of procaspase-8 to its active form, thereby initiating the caspase cascade leading to poly ADP-ribose polymerase (PARP) cleavage among others and eventually apoptosis [10].

Previous work done in our laboratory demonstrated that TULV infection induces apoptosis in Vero E6 cells and that externally added TNF-α enhances the cell death process [11]. To shed light on the molecular mechanisms which facilitate TNF-α mediated apoptosis in hantavirus-infected cells, we studied the activation of extracellular-signal regulated kinases 1 and 2 (collectively referred to as ERK1/2), a well-known group of mitogen-activated kinases (MAPKs) and regulators of cell survival. We now show that both apathogenic and HFRS-causing hantaviruses act in synergy with TNF-α to inactivate the ERK survival pathway.

## Results and discussion

### TULV inhibits ERK1/2 activity in Vero E6 cells

**Figure 1:**
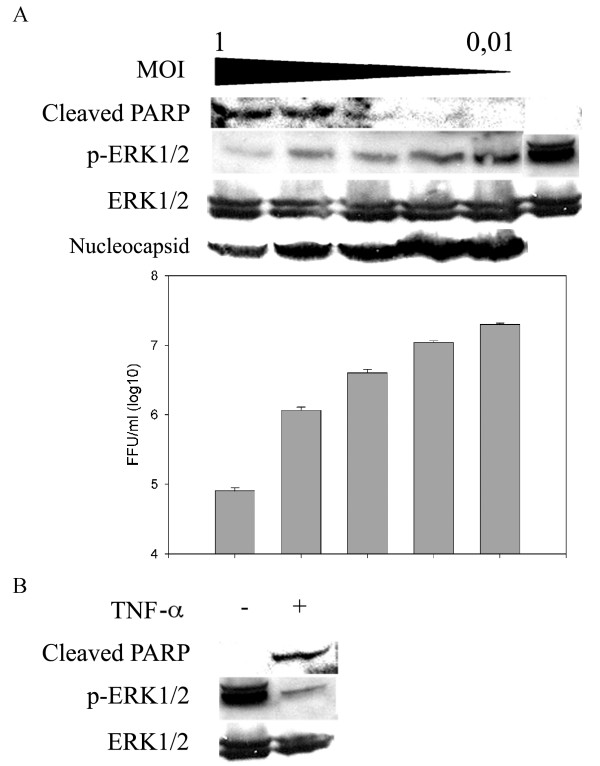
**TULV inhibits ERK1/2 cell survival pathway in Vero E6 cells**. A. In order to determine the relationship between TULV-induced apoptosis and ERK1/2 activity, Vero E6 cells were infected with 0.01, 0.1, 0.2, 0.5 or 1.0 multiplicity of infection (MOI) of TULV or mock-infected with fresh cell culture medium. B. Vero E6 were also treated (+) or non-treated (-) with a cell death-inducing concentration of TNF-α (100 ng/ml). Cells were collected at 11 days post infection or post TNF-α addition and 100 μg of protein lysate immunoblotted to detect cleaved PARP, phosphorylated ERK1/2 (p-ERK1/2), total ERK1/2 and hantavirus nucleocapsid protein N. Virus titers were determined as focus forming units (FFU) from conditioned media of infected cell cultures. Error bars for virus-titer measurements represent standard deviation. Experiments showing ERK1/2 dephosphorylation in TULV-infected cells are representative of multiple studies.

We studied the cellular signaling pathways which promote cell survival in hantavirus-infected cell cultures in order to get insight on the mechanisms behind hantavirus-induced apoptosis. We infected Vero E6 cells with Tula hantavirus and investigated the responses of one of the best-known cellular signaling mediators ERK1/2, the activation state of which is known to be regulated by phosphorylation [12]. We detected ERK1/2 proteins phosphorylated on tyrosine-204 by immunoblotting. Cells were infected with multiplicity of infection (MOI) between 1 and 0 of TULV or a cell death-inducing concentration of TNF-α. The cells were collected at 11 days post infection (p.i.), when cell death with the highest MOIs used was evident. We could confirm that increasing MOI resulted in higher degree of apoptosis, as judged by the amount of cleaved PARP (Figure 1A). In contrast to enhanced PARP cleavage, TULV infection resulted in a MOI-dependent reduction in phosphorylated ERK1/2 (p-ERK1/2) protein levels. The magnitude of ERK1/2 inhibition correlated directly with increasing MOI and apoptosis. However, we could also see ERK1/2 inhibition in cells where no apoptosis was detected (cells infected with MOIs 0.01 and 0.1). This implies that ERK1/2 inactivation is at least partially a direct cause of TULV infection and not solely an indirect event due to apoptosis. We also studied the amount of virus replication in infected cells by immunoblotting of the nucleocapsid protein and quantification of released infectious virus. Our results showed that virus replication was severely compromised in infected cells undergoing apoptosis (amount of released virus was decreased ~1000 times compared to viable cells). The treatment of Vero E6 cells with a high concentration of TNF-α resulted in a similar level of apoptosis and reduction of ERK1/2 activity compared to cells infected with 0.5 MOI of TULV (Figure 1B). This in turn suggested that the higher level of ERK1/2 inactivation which was seen in cells infected with MOIs from 1 to 0.2, as compared to lower MOIs used, was not only due to viral replication but also due to induced apoptosis. These results show that PARP cleavage in Vero E6 cells is accompanied by ERK1/2 inactivation and confirm that ERK1/2 activity is an important factor for maintaining cell viability.

**Figure 2:**
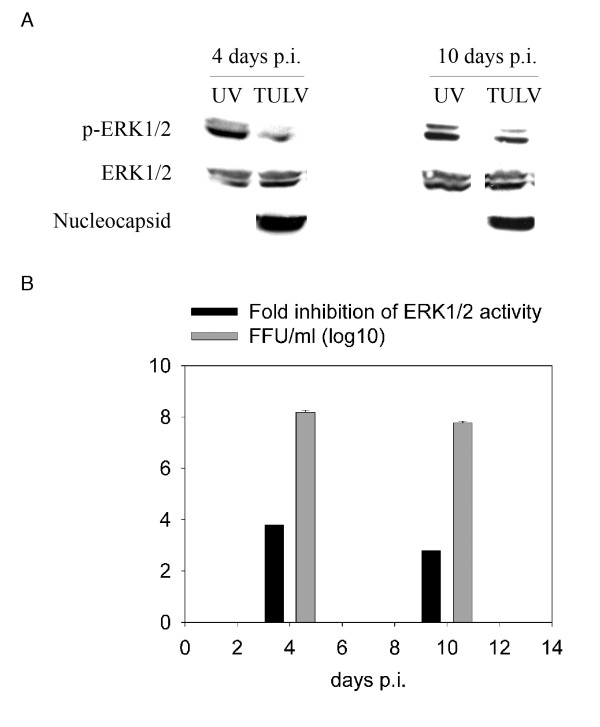
**TULV-induced ERK1/2 inactivation correlates with replication efficiency**. To confirm that TULV-mediated ERK1/2 inactivation is replication-dependent, we employed UV-inactivated TULV as a replication-incompetent negative control in ERK1/2 phosphorylation assay. Vero E6 cells were infected with a 0.1 multiplicity of infection of TULV or mock-infected with UV-inactivated virus (UV). Cells were collected at 4 and 10 days post infection and 100 μg of protein lysate immunoblotted to detect phosphorylated ERK1/2 (p-ERK1/2), total ERK1/2 and hantavirus nucleocapsid protein N. Bands were subjected to intensity analysis (ImageJ software; ) and the amount of p-ERK1/2 related to the amount of total ERK1/2 in individual samples. Fold change was calculated in relation to mock-infected sample at the respective day post infection (p.i.). Virus titers were determined as focus forming units (FFU) from conditioned media of cell cultures. Error bars for virus titer-measurements represent standard deviation.

To verify that ERK1/2 down-regulation was mediated by virus replication and not merely by adsorbed viruses or some other agents derived from infected cell culture supernatants, we used UV-inactivated TULV as a control in ERK1/2 phosphorylation analysis. Vero E6 cells were infected with non-treated or UV-inactivated TULV (MOI 0.1) for 4 and 10 days (Figure 2). We could confirm that TULV inhibited ERK1/2 phosphorylation as compared to UV-inactivated virus at both time points, indicating dependence on virus replication. Immunoblotting of the nucleocapsid protein and quantification of infectivity of released virus revealed that virus replication was relatively high already at 4 days p.i. (10^8 ^FFU/ml) and then decreased slightly at 10 days p.i.. Interestingly, replication efficiency correlated with the magnitude of ERK1/2 inactivation.

### HFRS-causing hantaviruses do not have the same capability as TULV to inhibit ERK1/2 activity

**Figure 3:**
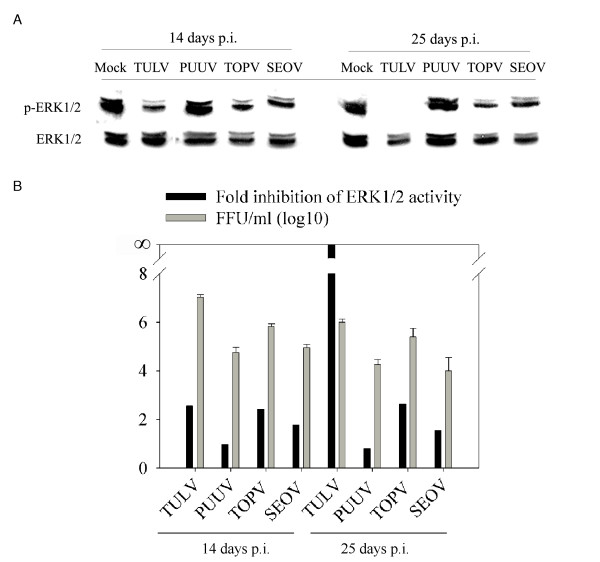
**HFRS-causing hantaviruses do not have the same capability as TULV to inhibit ERK1/2 activity**. To assess the ability of hantaviruses other than TULV to inhibit ERK1/2, Vero E6 cells were mock-infected with fresh cell culture medium or infected with TULV, PUUV, TOPV and SEOV at a multiplicity of infection of 0.01 for 14 and 25 days. Cell lysates (50 μg protein) were immunoblotted for detection of phosphorylated ERK1/2 (p-ERK1/2) or total ERK1/2 (A). Bands were subjected to intensity analysis (ImageJ software; ) and the amount of p-ERK1/2 related to mock sample at 14 and 25 days post infection. To investigate the correlation between ERK1/2 inhibition and the amount viral replication, virus titers were determined as focus forming units (FFU) from conditioned media of cell cultures and plotted together with fold inhibition of ERK1/2 activity in respective cells (B). Error bars for virus-titer measurements represent standard deviation. p.i. post infection.

Since Tula hantavirus is considered to be an apathogenic hantavirus we wanted to know whether pathogenic hantaviruses have the same capability as TULV to inhibit ERK1/2 activity. Hantaviruses are well known to replicate slowly in cell cultures, which might reflect the long incubation times of the virus seen also in HFRS patients [2]. We therefore incubated the infected cell cultures for up to 25 days p.i.. In addition to TULV, we used TOPV, an apparently apathogenic hantavirus, and SEOV and PUUV, two HFRS-causing hantaviruses. All hantaviruses had a minor or indiscernible negative effect on ERK1/2 activity at 14 days p.i. (Figure 3A). At 25 days p.i. ERK1/2 activity was almost totally abolished in TULV-infected cells whereas no dramatic changes, as compared to 14 days p.i., were seen with other hantaviruses studied. To compare the effect of virus growth rates on ERK1/2 activity, we measured virus titers from supernatants of the infected cells. We observed strikingly different amounts of virus released from cells infected with the different hantaviruses. The highest virus titers were obtained with TULV and were of the order of 10^7 ^FFU/ml, which is about ten to hundred times more than with other hantaviruses. The titers at 14 and 25 days p.i. are shown in Figure 3B, where they are compared with the magnitude of ERK1/2 inhibition. The amount of released virus correlated with the respective levels of ERK1/2 inhibition at 14 days p.i. for TULV, TOPV and SEOV. In PUUV-infected cells, which had the lowest virus production, we could not see any ERK1/2 inhibition. At 25 days p.i. the amount of released virus was generally lower than at 14 days p.i., possibly reflecting the deteriorated state of Vero E6 cell culture in terms of virus production after such a long period of incubation. The lower level of virus replication at this time point probably explains the lower level of ERK1/2 inhibition seen in TOPV- and SEOV-infected cells. Only in the case of TULV was ERK1/2 inhibition increased with simultaneous decrease in virus production. This might reflect the comparatively high amount of virus release from TULV-infected cells that could lead to an irreversible ERK1/2 inhibition due to apoptosis. Taken together, ERK1/2 inactivation by PUUV, TOPV and SEOV is directly correlated with virion production which suggests that there might exist a threshold level of hantavirus replication under which hantaviruses are still able to maintain host cell viability. However, an inherent difference in TULV among hantaviruses to cause marked ERK1/2 inactivation and apoptosis cannot be excluded.

### Hantaviruses and TNF-α act synergistically to inhibit ERK1/2 activity

**Figure 4:**
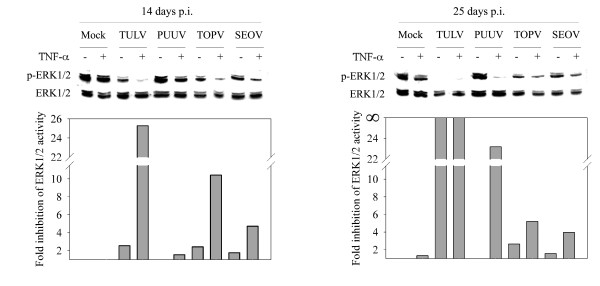
**Hantaviruses and TNF-α synergistically inhibit ERK1/2 activity**. To evaluate the role of TNF-α in hantavirus-mediated ERK1/2 inactivation, Vero E6 cells infected with different hantaviruses (see Figure 3) were incubated with (+) or without (-; same samples as in Figure 3) TNF-α (20 ng/ml). Fresh TNF-α was added together with fresh cell culture medium once a week. Cell lysates (50 μg protein) were immunoblotted for detection of phosphorylated ERK1/2 (p-ERK1/2) or total ERK1/2. Bands were subjected to intensity analysis (ImageJ software; ) and the amount of p-ERK1/2 related to mock sample without TNF-α treatment at 14 and 25 days post infection (p.i.).

Our previous results indicate that TNF-α augments TULV-induced apoptosis [11] and as TNF-α is considered to be an important factor in hantavirus pathogenesis, we wanted to evaluate its effect on hantavirus-mediated ERK1/2 inhibition. We incubated infected cells in the presence or absence of TNF-α and collected the cells concurrently (same samples as analyzed in Figure 3). Our results demonstrate that TNF-α acted in synergy with hantaviruses to inhibit ERK1/2 activity. The additional effects of TNF-α on ERK1/2 inhibition were from 2- to 20-fold (Figure 4). Interestingly, TNF-α could inhibit ERK1/2 also in PUUV-infected cells, where no ERK1/2 inhibition was seen by infection alone. Altogether, these results indicate that there are differences between hantaviruses in their ability to reduce ERK1/2 activity but that TNF-α has a general synergistic inhibitory effect on this pathway. Despite our efforts, even though these cells produce high amounts of virus, we could not detect any cleaved PARP by immunoblotting. This result implies that TULV-induced apoptosis is not directly associated with viral replication but is a consequence of a high MOI applied on cell culture. This in turn argues that also pathogenic viruses could cause apoptosis in Vero E6 cells if a high enough MOI is applied. However, because of their inability to replicate to similar high titers as TULV in these cells (see Figure 3), obtaining such high MOIs with pathogenic viruses was not feasible. In addition, our attempts to increase virus titers of pathogenic hantaviruses by ultracentrifugation have so far been unsuccessful. Also, as Vero E6 cells, to our knowledge, is the only cell type which promotes such a high replication-efficiency of hantaviruses, obtaining similar results as presented here with another commonly used cell line is unlikely.

## Conclusion

In characterization of the mechanisms of hantavirus-mediated apoptosis further, we demonstrated virus replication-dependent down-regulation of ERK1/2 by TULV, TOPV and SEOV, which was synergistically enhanced by TNF-α. ERK1/2 inhibition was induced by TNF-α also in PUUV-infected cells. ERK1/2 refers to prototype members of the mitogen-activated protein kinase (MAPK)-family that regulate cell proliferation, cell differentiation, cell cycle and cell survival [12]. ERK1/2 is activated by phosphorylation to threonine and tyrosine residues, which results in ERK1/2 translocation from the cytosol to the nucleus to regulate transcription. The ERK1/2 pathway is activated in many types of cancer and it promotes cell survival, i.e. it induces anti-apoptotic genes such as Bcl-2 and inactivates the pro-apoptotic Bad [13]. In addition, activation of the ERK1/2 pathway has been shown to protect cells from TNF-α-induced apoptosis [14,15]. ERK1/2 activity has been shown to be required for the efficient replication of many viruses [16-21]. In contrast, some viral proteins, like Ebola virus glycoprotein [22], hepatitis C virus non-structural protein NS5A [23], and human immunodeficiency virus (HIV) type 1 vpr protein [24] have been shown to down-regulate ERK1/2 activity. To our knowledge, however, our results are the first showing a direct virus replication-mediated down-regulation of ERK1/2 survival pathway in cell culture. Our results show a high basal ERK1/2 activity in confluent mock-infected Vero E6 cells that promotes cell survival even in the presence of sustained TNF-α treatment. However, in the infected cells ERK1/2 activity is reduced, which might at least in part render these cells sensitive to external TNF-α-mediated apoptosis. It would be of interest to understand the role of ERK1/2 activity in terms of viability of hantavirus-infected cells in more detail. Whether external activation of this pathway can rescue from hantavirus-mediated cell death remain to be answered.

The first evidence of hantavirus-induced apoptosis in cultured cells was described in Vero E6 cells with Hantaan virus, the prototype hantavirus to cause HFRS, and with Prospect Hill, an apparently apathogenic hantavirus [25]. Vero E6 cells are derived from monkey kidney epithelium and another kidney epithelial cell line, HEK-293, was later also shown to be susceptible to hantavirus-mediated apoptotic cell death [26]. Besides regulating apoptosis, ERK proteins have other essential roles in the kidneys. They promote tubular epithelial cell proliferation [27,28] and epithelial cell barrier resistance [29,30] thereby maintaining the integrity of a functional organ. Taken together with our previous work on TULV-induced apoptosis of Vero E6 cells [11,31] the present findings show that hantaviruses can hazard epithelial cell viability through apoptosis and ERK1/2 inactivation, at least in the presence of TNF-α.

In HFRS, one of the most prominent clinical manifestations is renal dysfunction leading to proteinuria. Kidney tubular epithelium degeneration and tubular epithelial cell death have been suggested to occur in PUUV-caused HFRS [32]. Also, hantaviral antigens have been detected in the renal tubular epithelial cells of HTNV- [33] and PUUV-infected patients [34]. Although epithelial cells may not be the main site of viral replication in man in the case of HFRS, viral replication in renal tubular epithelial cells could be the direct cause of renal epithelium dysfunction through direct virus-induced inhibition of signaling pathways necessary for cell viability (ERK1/2), which would be amplified by cytokines elevated in HFRS (TNF-α). Interestingly, Klingström et al. [35] showed recently an increase in the caspase cleavage product CK18, a marker for epithelial cell apoptosis, in sera of patients infected with PUUV. While the apathogen TULV also has the capacity to induce apoptosis and ERK1/2 inactivation in epithelial cells, one might rationalize that due to unidentified viral determinants apathogenic hantaviruses never make contact with the renal epithelium *in vivo *or are efficiently eliminated without causing notable renal symptoms or disease.

## Methods

### Viruses and cell cultures

TULV Moravia strain 5302, TOPV, SEOV and PUUV Sotkamo strain were propagated in Vero E6 cells in which they have been isolated and to which they are adapted producing titers of 10^4^-10^7 ^focus forming units (FFU)/ml conditioned medium [1,36,37]. Vero E6 cells (green monkey kidney epithelial cell line; ATCC: CRL-1586) were grown in minimal essential medium supplemented with 10% heat-inactivated fetal calf serum, 2 mM glutamine, 100 IU/ml of penicillin and 100 μg/ml of streptomycin, at 37°C in a humidified atmosphere containing 5% CO_2_. For the experiments, Vero E6 cell monolayers were grown to confluence, virus adsorbed for one hour at 37°C and growth medium added. For mock infections, either fresh culture medium or UV-inactivated virus was used. UV-inactivation was achieved using a stock of virus on ice in a lid-less 3 cm diameter culture dish, which was irradiated at 254 nm using a 30 W UV lamp at a distance of 10 cm with an exposure time of 30 min. The medium of infected and mock-infected cultures was changed once a week. In experiments where TNF-α was used, fresh TNF-α was added together with medium change. Viral titers in supernatants of infected cells were determined as described by Kallio et al. [38]. Briefly, 10-fold diluted supernatants were grown in Vero E6 cells on a 10-well microscopic slide and fluorescently stained for virus. Standard deviations were calculated from 4 individual wells. TULV-conditioned medium collected at 7 days p.i. and TOPV-, SEOV- and PUUV-conditioned media collected at 14 days p.i. were stored at -70°C and used as virus inocula.

### Antibodies and reagents

Mouse monoclonal antibody against phosphorylated form of ERK1/2 was from Santa Cruz Biotechnology Inc. Mouse monoclonal antibody against cleaved PARP and rabbit polyclonal antibody against ERK1/2 were from Cell Signaling Biotechnology. Rabbit polyclonal antibodies against Puumala hantavirus N have been described previously [39]. Recombinant human TNF-α was from R&D Systems.

### Immunoblotting

Infected and mock-infected Vero E6 cells (grown in 75-cm^2 ^or 25-cm^2 ^flasks) were scraped off into medium, washed twice with phosphate-buffered saline (PBS) and lysed in radioimmunoprecipitation (RIPA) buffer containing 50 mM Tris-HCl pH 7.5, 150 mM NaCl, 3 mM EDTA, 1% NP-40, 1 mM dithiothreitol (DTT), 1 mM Na_3_VO_4_, 20 mM NaF and EDTA-free cocktail of protease inhibitors (Roche). The protein concentrations of the cell lysates were determined using BCA Protein Assay Kit (Pierce). Laemmli gel loading buffer was added into samples, which were denatured at 95°C for 5 min and stored at -20°C. Samples were analyzed by immunoblotting according to standard protocols using 10% sodium dodecyl sulfate – polyacrylamide gel electrophoresis (SDS-PAGE).

## Competing interests

The authors declare that they have no competing interests.

## Authors' contributions

TS participated in the design of the study, performed the experiments and drafted the manuscript. JH analyzed data and participated in drafting the manuscript. HW participated in drafting the manuscript. AV participated in the design of the study and drafting the manuscript. HL designed the study and participated in drafting the manuscript. All authors read and approved the final manuscript.
